# Protocol for the Preparation of Polyacrylamide Hydrogels with Tunable Stiffness and Their Application in Cell Culture, Live‐Cell Imaging, and Fixed‐Cell Immunofluorescence

**DOI:** 10.1002/cpz1.70478

**Published:** 2026-10-19

**Authors:** Shatruhan Singh Rajput, Osian W. Jones, Laura M. Machesky

**Affiliations:** ^1^ Department of Biochemistry, Sanger Building, 80 Tennis Court Road University of Cambridge Cambridge UK

**Keywords:** cell fixation, confocal imaging, mechanosensing, polyacrylamide hydrogel, substrate stiffness

## Abstract

Tissue stiffness changes markedly in many pathological conditions, such as cancer and fibrosis, influencing cellular behavior and function. To investigate these effects *in vitro*, it is necessary to culture cells on substrates whose mechanical properties can be tuned to mimic both normal and diseased tissue environments. This protocol provides a detailed, reproducible procedure for preparing polyacrylamide (PAA) hydrogels with defined stiffness values that are functionalized with extracellular matrix (ECM) proteins and suitable for cell culture and imaging studies. It outlines the steps for cleaning and functionalizing glass coverslips, preparing acrylamide and bis‐acrylamide mixtures of desired concentrations, initiating polymerization, and conjugating ECM proteins to support cell adhesion. The protocol further describes the process of seeding cells onto the hydrogels, followed by live‐cell imaging, chemical fixation, and staining for visualization of cellular structures and protein localization. Practical notes and troubleshooting tips are also included for maintenance of hydrogel integrity and optimization of experimental reproducibility. The workflow enables systematic investigation of how substrate stiffness regulates cell morphology, migration, cytoskeletal organization, and other mechanosensitive responses. Overall, this protocol provides a reliable framework for preparing and using tunable PAA‐based hydrogels in studies of cell–matrix interactions, mechanotransduction, and disease‐related changes in tissue mechanics. It is adaptable to a wide range of cell types and compatible with various imaging modalities, offering a versatile platform for mechanobiology research. © 2026 The Author(s). *Current Protocols* published by Wiley Periodicals LLC.

**Basic Protocol 1**: Preparation of polyacrylamide‐based hydrogels of tunable rigidities and functionalization for cell culture

**Support Protocol 1**: Preparation of reagents for hydrogel preparation

**Basic Protocol 2**: Staining for live‐cell imaging and visualization of cellular structures and protein localization

**Basic Protocol 3**: Chemical fixation and immunofluorescence staining for visualization of cellular structures and protein localization

**Support Protocol 2**: Preparation of reagents and materials for cell fixation and immunostaining.

## INTRODUCTION

Cells actively explore and sense their environment and adjust their activity and organization to respond to external biochemical and mechanical cues. They are exposed to various external physical forces in physiological conditions, including tensile and compressive forces, while also applying their own forces to their environment and sensing the resulting response to actively probe environmental mechanical properties. These mechanical interactions are continuously integrated into cellular responses and regulate a range of processes including shape changes, migration, proliferation, and differentiation (Nikolaou & Machesky, 2020; Papalazarou et al., 2018).

The ability of a material to resist deformation under an applied force, referred to as stiffness but formally defined as Young's Modulus, is a key physical property of the extracellular environment that dictates cellular adaptations. As cells migrate through the body, they may encounter a large range of stiffnesses across different tissues and substantial variation in local stiffness within tissues, spanning many orders of magnitude. Soft tissues such as the brain (<1 kPa) and lung (1–8 kPa) present low stiffness conditions in the kilopascal range (Menal et al., 2018; Sicard et al., 2017). However, tissues such as muscle, cartilage, and bone possess progressively higher stiffnesses extending into the megapascal and gigapascal range (Bontempi et al., 2022; Ihnatouski et al., 2020). Tissue stiffness is also often altered in disease, with increased extracellular matrix (ECM) deposition and remodeling associated with increased stiffness in pathological conditions such as fibrosis and many forms of invasive cancer (Júnior et al., 2023; Massey et al., 2024). These changes to local stiffness can influence cellular organization and behavior, and as such, environmental stiffness is increasingly recognized as a key regulator of both healthy tissue function and disease progression.

Standard *in vitro* cell culture involves seeding cells on glass or plastic surfaces with a Young's modulus far exceeding that which cells typically encounter in physiological conditions. As such, these cells experience a mechanical environment that does not accurately reflect *in vivo* conditions. To study the influence of substrate stiffness on cell behavior within a physiologically relevant range, hydrogel‐based platforms have been developed. The stiffness of these biologically inert hydrogels can be carefully modified during preparation, and their surface can be functionalized with desired matrix proteins such as fibronectin or collagen to allow cell adhesion. In addition, their optical transparency makes them well suited for high‐resolution imaging.

Detailed protocols for the fabrication and characterization of polyacrylamide (PAA)‐based hydrogels have been described previously (Cresens et al., 2026; Tse & Engler, 2010). The method described by Cresens et al. focuses on the preparation of bulk, relatively thick PAA hydrogels (approximately 1.2–1.5 mm) with tunable stiffness values spanning approximately 1–60 kPa. The mechanical properties of these hydrogels are characterized using a stress‐controlled rotational rheometer with parallel‐plate geometry. This approach is particularly useful for applications such as cell culture followed by cell harvesting and fluorescence imaging performed from the cell‐facing surface. For applications requiring high‐resolution imaging through the hydrogel, however, thinner substrates are advantageous because the relatively large hydrogel thickness can limit imaging quality. In contrast, the method described by Tse & Engler et al. enables the preparation of thin PAA hydrogels suitable for high‐resolution imaging through the hydrogel. In that protocol, hydrogel stiffness was characterized by AFM nanoindentation using sharp, nanometer‐scale conical probes, with the reported hydrogel stiffness ranging from approximately 200 Pa to 35 kPa.

In this protocol, we describe a complementary and streamlined approach for preparing thin PAA hydrogels (approximately 200–300 µm thick) with tunable and defined stiffness values ranging from approximately 30 Pa (supersoft) to 40 kPa (Table 1). The method is designed to be straightforward and relatively time‐efficient while maintaining compatibility with a range of downstream applications, including high‐resolution imaging through the hydrogel, AFM nanoindentation measurements, and cell culture for applications such as transcriptomic and other omics analyses. Importantly, the mechanical properties measured by AFM nanoindentation can depend on experimental parameters including indenter geometry and size, indentation depth, sample thickness, and the contact‐mechanics model used for data analysis (Costa & Yin, 1999; Huth et al., 2019). Careful consideration of these parameters is therefore important when characterizing PAA hydrogels for quantitative cell mechanobiology studies.

**Table 1:** Young's Modulus Measured Using an Atomic Force Microscope Equipped with a Customized Tipless Cantilever Modified with a 5‐µm‐Diameter Glass Bead

Mixing order	Material	0.028 ± 0.01 kPa (µL)	0.087 ± 0.04 kPa (µL)	0.25 ± 0.08 kPa (µL)	1.3 ± 0.1 kPa (µL)	7.5 ± 0.9 kPa (µL)	36.0 ± 12 kPa (µL)
1	Milli‐Q Water	**827.5**	**822.5**	**812.5**	**777.5**	**642.5**	**517.5**
2	AAm	**60**	**70**	**75**	**90**	**125**	**250**
3	Bis‐AAm	**30**	**25**	**30**	**50**	**150**	**150**
4	TEMED solution	**62.5**	**62.5**	**62.5**	**62.5**	**62.5**	**62.5**
5	APS solution	**20**	**20**	**20**	**20**	**20**	**20**

Hydrogel stiffness is characterized using a customized tipless AFM cantilever functionalized with a 5‐µm‐diameter glass bead (Singh et al., 2024). The spherical probe provides a microscale contact geometry that is relevant to the length scale over which adherent cells mechanically interact with their underlying substrate. Hydrogel stiffness is determined from AFM nanoindentation measurements using the Hertz contact model, yielding stiffness values within the intended range for cell mechanobiology studies. The use of a microscale spherical probe provides a practical and reproducible approach for characterizing the mechanical properties of PAA hydrogels at a length scale relevant to cell–substrate interactions and focal adhesion–mediated mechanotransduction.

Established protocols describe the preparation of tunable PAA hydrogels (Cresens et al., 2026; Tse & Engler, 2010); however, we found several steps could be omitted without compromising hydrogel quality, production, or mechanical properties. This protocol does not include glutaraldehyde crosslinking to enhance hydrogel–glass adhesion as previously described (Tse & Engler, 2010) because the 3‐aminopropyltrimethoxysilane (APTMS) activation of the glass surface provides sufficient adhesion to prevent delamination of thin PAA hydrogels. Some protocols include a release‐surface treatment to facilitate removal of the coverslip after polymerization; however, we found that thin gels detach cleanly and reproducibly without this step. These gels retained optical clarity without degassing, and AFM measurements across multiple positions (n ≈ 20) on all hydrogel surfaces showed low spatial variability in mechanical stiffness (≈12% relative standard deviation), confirming uniform polymerization.

We describe detailed procedures for preparing mammalian cells for live‐cell imaging and immunofluorescence experiments on PAA hydrogels. Basic Protocol 1 describes the preparation of PAA hydrogels with tunable stiffness and their functionalization with fibronectin. Basic Protocol 2 outlines procedures for live‐cell staining and imaging of cells cultured on PAA hydrogel substrates, enabling visualization of dynamic cellular processes in response to controlled changes in substrate stiffness. Basic Protocol 3 describes chemical fixation and immunofluorescence staining of mammalian cells cultured on PAA hydrogels. Together, these protocols provide a reliable and accessible experimental platform for investigating how substrate stiffness influences cell behavior, cytoskeletal organization, and mechanotransduction across physiologically relevant mechanical regimes.

## STRATEGIC PLANNING

The complete preparation and functionalization of PAA hydrogels with ECM proteins typically require approximately 24 hours. This includes hydrogel polymerization, surface activation, and overnight incubation with ECM proteins to facilitate cell adhesion. After 24 hours, cells can be seeded onto the functionalized hydrogels and cultured for the desired duration. For downstream analysis, cells may be subjected either to live‐cell immunostaining, which requires approximately 30 minutes of incubation with fluorescent probes, or to fixation followed by immunofluorescent staining for subsequent imaging. The fixation and staining steps together typically take 4–5 hours and can be completed on the same day. Fixed and stained samples can be stored for future imaging as needed. We did not observe any significant changes in hydrogel stiffness after 3 weeks of storage in PBS at 5 °C.

## Basic Protocol 1 PREPARATION OF POLYACRYLAMIDE (PA) HYDROGELS

This protocol provides a streamlined and complementary approach to previously reported methods for the preparation of PAA hydrogels with tunable stiffness (Tse & Engler, 2010). While the previously reported protocol includes (NaOH‐specific) selected steps performed at 80°C, the steps described here are carried out at ambient temperature and involve fewer processing steps. This simplified workflow facilitates efficient and reproducible preparation of PAA hydrogels while maintaining consistent hydrogel quality. When the procedures are followed carefully, the method routinely produces flat, uniformly polymerized, and well‐adhered hydrogels with a high preparation success rate. In our experience, when preparing batches of approximately 10 hydrogels, typically 9–10 hydrogels are suitable for subsequent experiments. An exception is the preparation of supersoft hydrogels (<100 Pa), for which the preparation success rate can be somewhat lower, reflecting the increased sensitivity of these very soft gels to handling and preparation conditions.

In addition, we provide optimized acrylamide and bis‐acrylamide compositions for obtaining a broad range of hydrogel stiffnesses, including supersoft hydrogels with stiffnesses of a few tens of pascals, intermediate‐stiffness hydrogels ranging from a few hundred pascals to a few kilopascals, and higher‐stiffness hydrogels in the several‐kilopascal range. Together, these features provide a practical and versatile approach for generating PAA hydrogels with defined mechanical properties across a broad stiffness range for cell biology and mechanobiology applications.

The hydrogel is prepared on glass substrates, such as glass coverslips for fixed samples, and Petri dishes with glass bottoms or inserted coverslips for live‐cell imaging. First, the glass surface is treated with NaOH to remove organic contaminants and increase the number of hydroxyl groups (‐OH) on the glass surface by slightly etching it. Then the surface is treated with APTMS, which reacts with the ‐OH groups and forms strong covalent Si–O–Si bonds with the glass, while amine groups stick out, which will further bind to the hydrogel. The hydrogel components are mixed in the appropriate ratio to achieve the desired substrate stiffness. A drop of the hydrogel mixture is then placed on the functionalized glass surface, followed by placing a clean glass coverslip on top of it and allowing it to polymerize. After polymerization, the top coverslip is carefully removed, and a hydrogel with a flat‐top surface is ready. The hydrogel is then treated with Sulfo‐SANPAH, a heterobifunctional crosslinker, which reacts on one side with the hydrogel and on the other side with ECM proteins such as fibronectin and collagen. Next, the hydrogel is treated with fibronectin (or collagen or other matrix proteins as per the requirement), making the hydrogels ready for cell culture. Now, cells can be seeded on this functionalized hydrogel substrate.

In the current protocol, we provide a list of necessary reagents and a protocol to prepare hydrogels with various rigidities. We use two rigidities (∼0.3 kPa and ∼35 kPa) as an example and treat the hydrogels with fibronectin and seed pancreatic ductal adenocarcinoma (PDAC) cells for fixed‐cell immunofluorescence staining and imaging, and for live‐cell fluorescence imaging using fluorescent proteins or fluorescent dyes.

### Materials

NaOH, 1M (Sigma‐Aldrich, cat. no. 06203‐1kg; see Support Protocol 1)
Milli‐Q water
(3‐aminopropyl)trimethoxysilane (APTMS; Sigma‐Aldrich, cat. no. 281778‐100ML)
Ice
Acrylamide, 40% (Sigma‐Aldrich, cat. no. A4058‐100ML)
Bis‐acrylamide, 2% (G Biosciences, cat. no. 786‐059)
TEMED (Sigma‐Aldrich, cat. no. T22500‐100ML; see Support Protocol 1)
Ammonium persulphate (APS; Severn Biotech Ltd, cat. no. 20‐3001‐01; see Support Protocol 1)
Dulbecco's Phosphate Buffered Saline (Sigma Aldrich, cat. no. D8537‐500ML)
Sulfo‐SANPAH (Thermo Fisher Scientific, cat. no. 22589; see Support Protocol 1)
Fibronectin (Sigma Aldrich, cat. no. F1141‐2MG; see Support Protocol 1)
Cultured cells

Glass coverslip, 19 mm (Agar Scientific, cat. no. AGL46R19‐0)
Plate, 12‐well
Glass coverslip, 16 mm (Academy, cat. no. NPC13/16)
Petri dish, 35 mm with 24‐mm glass bottom (World Precision Instruments, cat. no. FD35‐100)
UV light source, 365 nm
Fume hood
Weighing machine
Vortex mixer
Plastic tubes, 1.5 mL and 15 mL
Pipettes and tips, 20 µL, 200 µL, and 1000 µL
Timer
Beakers
Aluminum foil
Forceps (Dumont, cat. no. 11251‐35)
Syringe (Bicton Dickinson, cat. no. 307727)
Needle (TERUMO, cat. no. NN‐2138R)
Filter, 0.2 µm (Merck Millipore Ltd., cat. no. SLGS033SS)

#### Preparation of hydrogel

1 Add 1 mL of 1M NaOH solution onto the glass substrate and let it stand for 1 hour.
  a. *For Petri dishes with glass bottoms*: Apply the NaOH solution directly onto the glass surface.
  b. *For glass coverslips*: Place the 19‐mm coverslips on a 12‐well plate (one coverslip per well) and then add the NaOH solution.
2 Rinse three times with distilled water and let them completely dry (∼15–20 minutes at room temperature).
  a. *Petri dish*: Add ∼2 mL of distilled water, gently swirl to rinse the surface, and discard. Repeat this step three times.
  b. *Coverslips placed in a 12‐well plate*: Add ∼2 mL of distilled water directly to each well, gently swirl to rinse, and remove the liquid by tilting the plate or aspirating with a vacuum aspirator. Repeat this step three times.
  c. Place the substrate at a slight angle to allow residual water to drain off, promoting faster drying and preventing the formation of water patches.
3 Add 200 µL APTMS (bind‐silane) on glass and let it stand for 7 minutes.
  Exercise caution to prevent bind‐silane from contacting plastic surfaces, as it may chemically etch the material, resulting in surface damage and potential microcontamination.
4 Rinse three times with distilled water and let it dry completely (∼15–20 minutes at room temperature).
**For Petri dishes with glass bottoms**: Immerse the Petri dish(es) in a beaker of distilled water for 2–3 minutes. Replace it with fresh distilled water. Hold the dish in your hand while it remains submerged and shake it vigorously to remove residual bind‐silane. Inspect the glass surface to ensure the viscous silane layer is largely removed before proceeding.
**For coverslips**: Add distilled water to each well and let stand for 2–3 minutes. Replace it with fresh distilled water. Using forceps, remove individual coverslips from the wells, immerse them in distilled water, and shake vigorously to remove residual bind‐silane. Repeat this washing step three times. Inspect the coverslip surfaces to ensure that the viscous silane layer is largely removed before proceeding.
  Place the individual Petri dishes and coverslips at a slight angle to allow residual water to drain off, promoting faster drying and preventing the formation of water patches.
**For coverslips**: Place them on a parafilm sheet with the functionalized side facing upward and proceed with the next steps.
5 Prepare the hydrogel mixture and add 25 µL to the glass surface of a Petri dish (20 µL for a coverslip; see Table 1 for preparation details).
  a. **Follow the order of mixing the components carefully**: After combining Milli‐Q water, acrylamide (AAm), and bis‐acrylamide (Bis‐AAm), add TEMED first, followed by APS.
  b. Prepare the mixture in an ice bath to maintain a low temperature and prevent premature polymerization.
  c. After adding all components, mix thoroughly using a pipette. Use the solution immediately after adding TEMED and APS, as polymerization begins rapidly.
  d. Adjust the volume of the hydrogel mixture during casting to control the final thickness.
6 Place a prepared 19‐mm coverslip on top of the droplet on the glass surface of a Petri dish (16 mm for coverslips) and allow it to polymerize for 30 minutes.
  Clean the coverslip to be placed on top by wiping it with ethanol or briefly immersing it in ethanol to remove any dirt or contaminants.
7 Following 30 minutes of incubation, gently lift and remove the top coverslip with forceps, taking care not to disturb the hydrogel surface.
  a. Add 1 mL of DPBS (or Milli‐Q water) before removing the top coverslip to prevent it from falling back onto the hydrogel, as it will float on the liquid.
  b. After removing the top coverslip, inspect it to ensure it is clean and free of any hydrogel residue. If the hydrogel remains on the coverslip, the hydrogel surface will be rough and unsuitable for use.
8 Rinse the hydrogel three times with DPBS to remove residual debris.
  The hydrogel is now prepared and ready to be coated with ECM proteins to support cell culture.
9 Add 200 µL of Sulfo‐SANPAH solution (0.2 mg/mL) to the hydrogel surface and irradiate under UV light (365 nm) for 15 minutes.
  a. Thoroughly vortex the Sulfo‐SANPAH to dissolve it completely, and filter through a 0.2‐µm filter to remove large crystals that might stick to the hydrogel.
  b. If a dedicated UV source is unavailable, use the UV light inside a tissue culture hood and place the hydrogel as close to the light source as possible.
10 Rinse the hydrogel three times with DPBS to remove residual debris.
11 Add 1 mL of fibronectin (10 µg/mL) to the hydrogel and incubate overnight at 37 °C in a 5% CO_2_ incubator.
  Alternatively, you can coat the hydrogel with collagen instead of fibronectin, depending on the requirements of your experiment.
12 Following overnight incubation, wash the hydrogels by rinsing three times with DPBS to remove unbound protein.
**The hydrogel is now ready for cell culture, and cells can be directly seeded onto its surface**. You may use it immediately or store it by covering it with DPBS and sealing the container with parafilm. Be aware that extended storage can affect its mechanical properties.
  (Refer to Fig. 1 for representative images of hydrogels prepared on a Petri dish and a coverslip.)

**Figure 1:**
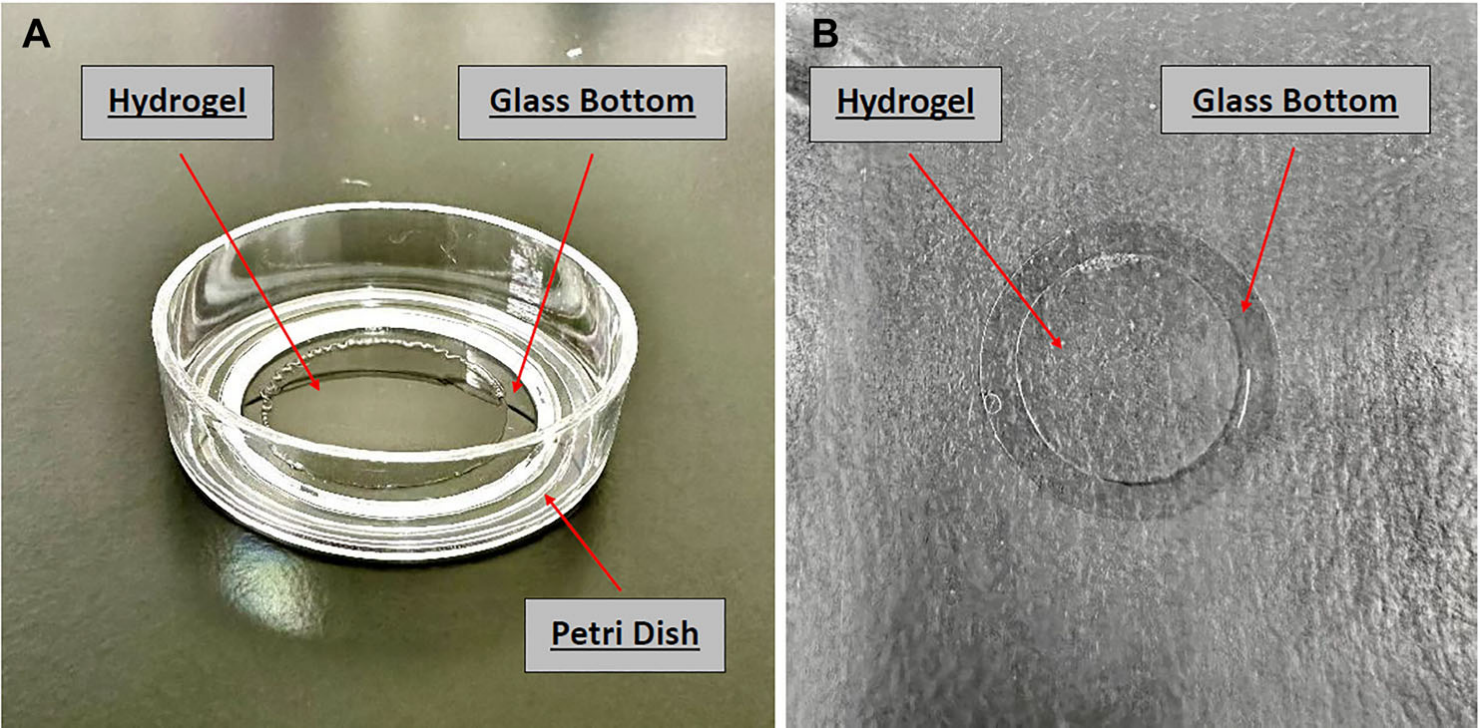
Pictures of hydrogel prepared on a Petri dish (left) and a glass coverslip (right). For samples cast in the Petri dish, the substrate (glass) diameter is 23 mm, and the hydrogel diameter is 19 mm. For samples cast on the glass coverslip, the substrate (glass) diameter is 19 mm, and the hydrogel diameter is 16 mm.

## Support Protocol 1 PREPARATION OF REAGENTS FOR HYDROGEL PREPARATION

In the hydrogel preparation protocol described above, certain reagents need to be prepared at specific concentrations or compositions. The following steps provide detailed instructions for their preparation.

### Materials

NaOH crystals
Sterile autoclaved Milli‐Q Water
TEMED
APS powder
Sulfo‐SANPAH
Fibronectin
DBPS
Acrylamide, 40%
Bis‐acrylamide, 2%

#### Preparation of 1M NaOH

1 Dissolve 40mg of NaOH crystals in 1 mL Milli‐Q water to obtain a 1M NaOH solution.

#### Preparation of TEMED solution

2 Add 12 µL of TEMED to 735 µL of Milli‐Q water and mix thoroughly by pipetting or vortexing. Perform this step under a fume hood.

#### Preparation of APS solution

3 Add 40 mg of APS powder to 760 µL of Milli‐Q water and mix thoroughly by pipetting or vortexing.

#### Preparation of hydrogel mixture

4 Follow the procedure in Table 1 to prepare the hydrogel mixture to achieve substrates with tuned rigidities. See Table 1 for use of Acrylamide, 40% (AAm) and Bi‐sacrylamide, 2% (BisAAm).

#### Preparation of Sulfo‐SANPAH solution

5 Dissolve 1 mg of Sulfo‐SANPAH powder in 1 mL of Milli‐Q water to prepare a 1 mg/mL stock solution and store it at −20 °C for future use. Dilute the stock solution fivefold (e.g., mix 1 mL of the 1 mg/mL stock with 4 mL of Milli‐Q water) to obtain a 0.2 mg/mL working solution.

#### Preparation of fibronectin solution

6 Dilute the fibronectin stock solution (1 mg/mL) 100‐fold in DPBS to obtain a working concentration of 10 µg/mL. For example, add 10 µL of the stock fibronectin to 1 mL of DPBS.

## Basic Protocol 2 LIVE‐CELL IMAGING

Live‐cell imaging enables the visualization of dynamic processes in real time, including changes in cell migration, morphology, and organelle organization. Imaging viable cells on PAA‐treated surfaces allows spatiotemporal observation of cellular events in response to carefully controlled substrate stiffnesses.

Cells are seeded onto fibronectin‐coated hydrogels and maintained under standard culture conditions until ready for imaging at 4–6 hours after seeding, up to 48 hours. Cells may be seeded at varying densities and time points depending on the experimental questions. Where required, cells expressing or labelled with fluorescent markers are imaged using the appropriate imaging platform and conditions.

### Materials

Cells of interest
Dulbecco's Phosphate Buffered Saline (DPBS; Sigma Aldrich, cat. no. D8537‐500ML)
MitoTracker Green FM (Thermo Fisher Scientific, cat. no. M7514; see recipe)
Leibovitz's L‐15 medium (Sigma Aldrich, cat. no. L5520‐ 100ml; see recipe)
Dulbecco's Modified Eagle Medium (DMEM) supplemented with 10% FBS, 1% L‐glutamine, and 1% Penicillin‐Streptomycin (Gibco, cat. no. 21969035; see recipe)

Incubator set to 37°C with 5% CO_2_
Pipettes and tips

#### Live‐cell imaging

1 Grow cells in Dulbecco's Modified Eagle Medium supplemented with 10% FBS (see recipe), 1% Lglutamine (see recipe), and 1% Penicilli‐nStreptomycin (see recipe) at 37°C and 5% CO_2_. Prior to cell seeding, rinse the hydrogel surface three times with DPBS to remove residual debris.
2 Prepare a single‐cell suspension at the appropriate concentration for the desired cell confluency.
  a. Add approximately 1 mL of the cell suspension to the hydrogel surface.
  b. Gently swirl the dish to ensure even cell distribution.
3 Transfer the dish to an incubator maintained at 37°C with 5% CO_2_ to allow attachment.
4 Cells may be imaged as early as 4–6 hours after seeding, once stable adhesion is established.
  Optional: If fluorescent dyes are required, add them according to the manufacturer's instructions or established methods. This step is typically performed immediately prior to imaging to minimize toxicity and ensure reporter performance.
  (Example: Cells were incubated in MitoTracker Green FM (1/1000 dilution in cell culture media) for 30 minutes at 37°C, washed once with fresh medium.)
5 Image cells directly in desired culture media using appropriate microscopy settings.
  a. If imaging in an incubator without CO_2_, Leibovitz's L‐15 medium (see recipe) should be used to maintain physiological pH levels.
    (Refer to Fig. 2 for representative images of PDAC‐A cells cultured on soft and stiff hydrogels.)

**Figure 2:**
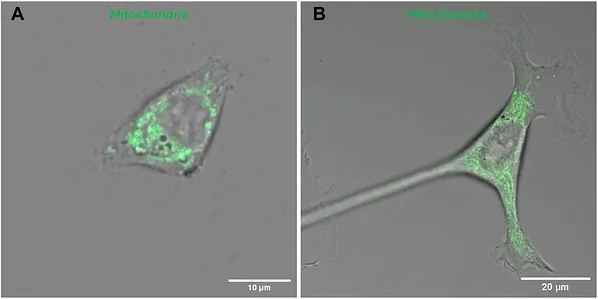
Confocal images of live PDAC‐A cells seeded on soft (left) and stiff (right) hydrogels for 24 hours and stained with MitoTracker Green FM for 1 hour. Bright‐field (grey) and the MitoTracker (green) channels are merged for composite visualization. Samples were imaged using a Zeiss LSM 980 confocal microscope.

## Basic Protocol 3 FIXATION AND IMMUNOSTAINING

Cell fixation is essential for preserving cellular morphology at a defined time point and enabling immunostaining to visualize intracellular structures with high resolution. Fixation and cell permeabilization allow the use of fluorescent dyes and antibodies that are not compatible with live‐cell imaging, such as DAPI for nuclear staining and phalloidin for visualizing filamentous actin. Fixation also permits stained samples to be stored for subsequent imaging. Under the storage conditions used in this protocol, we did not observe any significant changes in staining patterns or fluorescence intensity after 6 months of storage at 4°C.

In brief, cells were fixed with formaldehyde to chemically crosslink proteins to preserve cellular structures. The cells were then permeabilized using a permeabilization buffer to allow antibodies to enter the cell and bind to targets and subsequently stained with the desired chemical probes or antibodies. A BSA‐based blocking buffer was used to minimize non‐specific antibody binding.

In this protocol, we describe the fixation and staining steps to visualize filamentous actin (phalloidin) and nuclear DNA (DAPI), which do not require primary antibody binding. However, the same workflow can be applied for immunostaining with primary and secondary antibodies, involving sequential incubation with the primary antibody followed by the appropriate fluorescent secondary antibody. Additional details are provided in Support Protocol 2.

### Materials

Formaldehyde, 4% (Electron Microscopy Sciences, cat. no. 15710)
Permeabilization buffer (see Support Protocol 2)
Bovine Serum Albumin, 0.2% (BSA; Roche Diagnostics, cat. no. 10735108001)
Phalloidin‐AF 488 Phalloidin (Life Technologies Corporation, cat. no. A12379)
Mounting medium
PBS (regular, autoclaved)
DAPI (Abcam, cat. no. AB104139)
Ice

Fume hood
Glass coverslip, 16 mm (Academy, cat. no. NPC13/16)
Glass coverslip, 19 mm (Agar Scientific, cat. no. AGL46R19‐0)
Aluminum foil
Humidity chamber (see Support Protocol 2)
Glass slide (Epredia, cat. no. J1800AMNZ)
Plastic tubes, 1.5 mL and 15 mL
Timer
Beakers

#### Cell fixation and immunostaining

##### Fixation

1 Aspirate the culture medium and add 1 mL of 4% formaldehyde (see Support Protocol 2). Let it stand for 20 minutes.
  a. The sample (cells grown on hydrogel) is prepared on a glass coverslip and placed in a multiwell plate for cell culture purposes.
  b. Perform this step in a fume hood.
  c. Cover the plate with aluminum foil to protect from light damage for this and all following steps.
2 Remove the formaldehyde and rinse the sample three times with regular autoclaved PBS.
  Dispose formaldehyde in an appropriate chemical waste container.
  After this step, carry out all subsequent procedures outside the fume hood.

###### Permeabilization

3 Add permeabilization buffer (see Support Protocol 2) and let it stand for 15 minutes.
4 Rinse the sample three times with 0.2% BSA (see Support Protocol 2).
5 Place a piece of Parafilm in a humidity chamber (see Support Protocol 2) and apply approximately 50 µL of the phalloidin mixture (see Support Protocol 2).

###### Staining with phalloidin

6 Carefully position the sample hydrogel‐side down onto the phalloidin mixture and incubate for 20 minutes.
  Meanwhile, take a clean standard rectangular glass slide (25 × 75 × 1 mm), place it in a chamber covered with aluminum foil, and add approximately 40 µL of mounting medium onto the slide.
7 Rinse the sample three times with 0.2% BSA, followed by three washes with PBS.
  After rinsing, gently tap the edge of the coverslip on a paper towel to remove excess solution, and then proceed immediately to the next step.
8 Carefully position the sample hydrogel‐side down onto the mounting medium.
  Note that the mounting medium includes DAPI, eliminating the need for separate nuclear staining.
9 Keep the sample at room temperature for at least 2 hours, and then store it in the refrigerator at 4 °C.
  Following overnight incubation, seal the coverslip edges with nail polish to protect the sample from dehydration.
**The prepared sample is now ready for microscopic imaging**.
  (Refer to Fig. 3 for representative images of fixed PDAC‐A cells cultured on soft and stiff hydrogels.)

**Figure 3:**
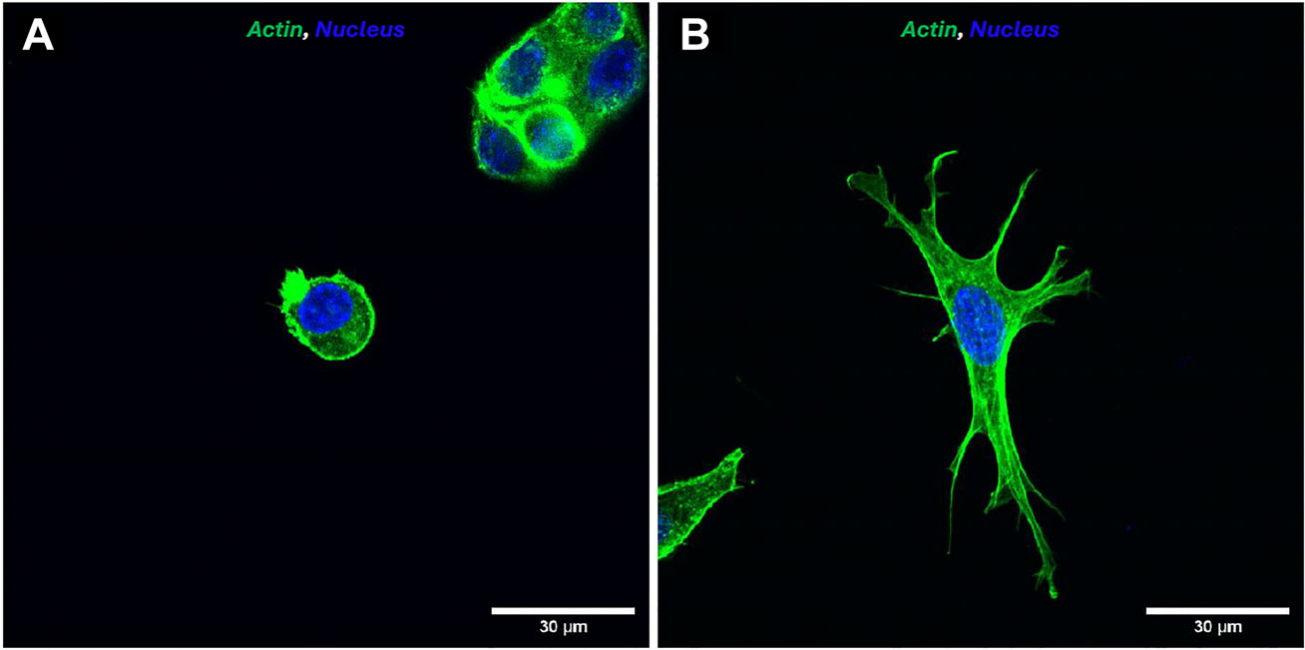
Representative confocal microscopy images of fixed PDAC‐A cells cultured on soft (left) and stiff (right) substrates. F‐actin was stained with Phalloidin Alexa Fluor 488 (green), and nuclei were counterstained with DAPI (blue). Samples were imaged using a Zeiss LSM 980 confocal microscope.

## Support Protocol 2 PREPARATION OF REAGENTS AND MATERIALS FOR CELL FIXATION AND IMMUNOSTAINING

In the cell fixation and immunostaining protocol described above, certain reagents need to be prepared at specific concentrations or compositions. The following steps provide detailed instructions for their preparation.

### Materials

Same as described in Basic Protocol 3.

#### 4% formaldehyde

1 The product described in the Materials section is 16%. Make it 4% by adding 1 mL of 16% formaldehyde to 3 mL of PBS.

#### 0.2% BSA

2 Add 1g BSA to 500 mL of PBS and dissolve it completely.

#### Permeabilization buffer (0.05% Triton)

3 Add 5 µl Triton X‐100 to 10 mL of 0.2% BSA and mix them thoroughly.

#### Phalloidin mixture

4 Dilute phalloidin 200 times or follow the manufacturer's instructions. Add 1 µl phalloidin to 200 µl PBS and mix well. Cover the vial with aluminum foil.

#### Humidity chamber

5 Take a plastic square plate (approximately 12 × 12 cm²) and cover both the base and lid with aluminum foil. Place wet tissue paper (roll up some tissue paper and moisten it with water) along the edges to maintain humidity inside the chamber. (Refer to Fig. 4 for a representative picture of a humidity chamber.)

**Figure 4:**
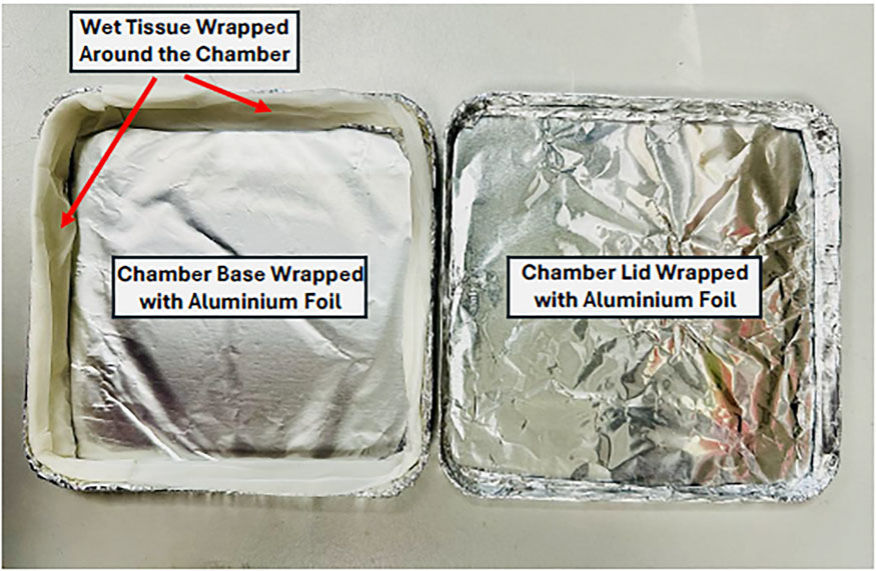
Picture of a humidity chamber. The base and lid are wrapped with aluminum foil, and wet tissue paper is placed around the internal base edges.

## REAGENTS AND SOLUTIONS

*Use Milli‐Q water in all recipes and protocol steps*.

### Live‐cell imaging

1× Dulbecco's Modified Eagle Medium, high glucose, with sodium pyruvate, without L‐glutamine (500 mL; 2–8°C; Gibco, cat. no. 21969035)
10% (v/v) final Fetal Bovine Serum, heat‐inactivated, non‐USA origin, sterile‐filtered (50 mL; −20 °C; Sigma‐Aldrich, cat. no. F9665‐500ML)
2 mM L‑Glutamine, 200 mM stock solution (5 mL; −20°C; Gibco, cat. no. 25030024)
10,000 U/mL Penicillin–Streptomycin solution [5 mL, 10,000 µg/mL streptomycin; 1% (v/v) final; −20°C; Gibco, cat. no. 15140122]
1× Leibovitz's L‑15 Medium (1 mL; 37°C; Merck–Sigma Aldrich, cat. no. L5520‐100ML)
1/1000 MitoTracker Green FM (1 µL; 37°C incubation; Thermo Fisher Scientific, cat. no. M7514)
1% (v/v) Penicillin–Streptomycin solution (10,000 U/mL penicillin, 10,000 µg/mL streptomycin; 5 mL; −20°C; Gibco, cat. no. 15140122)

## COMMENTARY

### Critical Parameters

Critical parameters requiring attention have been highlighted in the relevant steps of the protocol above.

### Troubleshooting Table

Tables 2 and 3 present the problems that may occur when performing the protocols and the possible solutions.

**Table 2:** Troubleshooting Guide for Hydrogel Preparation

Problem	Possible Cause	Solution
Distorted hydrogel surface and induced directional stress, leading to uneven stiffness or surface irregularities	Shearing the top coverslip during removal	Carefully lift the coverslip vertically upward using forceps instead of shearing it sideways.
Debris on the hydrogel surface interfering with cell attachment, imaging quality, and uniform coating of ECM proteins	1. Plastic residues etched from the dish edges during Bind‐Silane treatment 2. Undissolved Sulfo‐SANPAH crystals remaining in the solution	1. Thoroughly rinse the coverslip or dish to remove any plastic residues generated during the Bind‐Silane treatment. 2. Vortex the Sulfo‐SANPAH solution thoroughly and filter it through a 0.2‐µm filter before use to remove undissolved crystals.
Microbial contamination (bacterial or fungal growth)	Non‐sterile handling of the hydrogel after UV treatment	1. Handle the hydrogel carefully using sterile tools and gloves immediately after UV treatment. 2. Work within a clean laminar flow hood.

**Table 3:** Troubleshooting Guide for Fixing and Immunostaining

Problem	Possible Cause	Solution
Cell detachment	Harsh rinsing	Rinse the sample gently to avoid dislodging cells.
Cell damage	Pressing the sample against a rigid surface	Handle the sample carefully and place it gently, especially when positioning upside down.
Sliding sample or drying	Excess or insufficient mounting medium	Optimize the volume of the mounting medium according to the sample size to prevent sliding or drying.

### Understanding Results

#### Hydrogel

Representative images of hydrogels prepared on a Petri dish and a glass coverslip are shown in Figure 1. The diameters of the hydrogels formed on the Petri dish and glass coverslip are 19 mm and 16 mm, respectively.

#### Live‐cell images

Confocal images of live PDAC‐A cells cultured on soft and stiff hydrogel substrates are shown in Figure 2. Bright‐field (grey) and MitoTracker (green) channels were merged to generate composite images for visualization.

#### Fixed cell images

Confocal images of fixed PDAC‐A cells cultured on soft and stiff substrates and stained for F‐actin and nuclei are shown in Figure 3. The F‐actin and nuclear channels were merged to generate composite images for visualization.

#### Humidity chamber

A representative image of the humidity chamber used for the experiment is provided as a guide for its easy preparation in the laboratory (Figure 4). The exterior surfaces of both the base and lid were wrapped with aluminum foil to protect the samples from photobleaching caused by exposure to ambient light during stain incubation. Wet tissue paper was placed around the internal base edges.

#### Cell projected area

We quantified the mean spread area of fixed PDAC‐A cells cultured on soft and stiff substrates (Figure 5). A significant increase in projected cell area was observed on stiff substrates compared to soft substrates. This indicates that PDAC‐A cells exhibit distinct morphological phenotypes depending on substrate stiffness.

**Figure 5:**
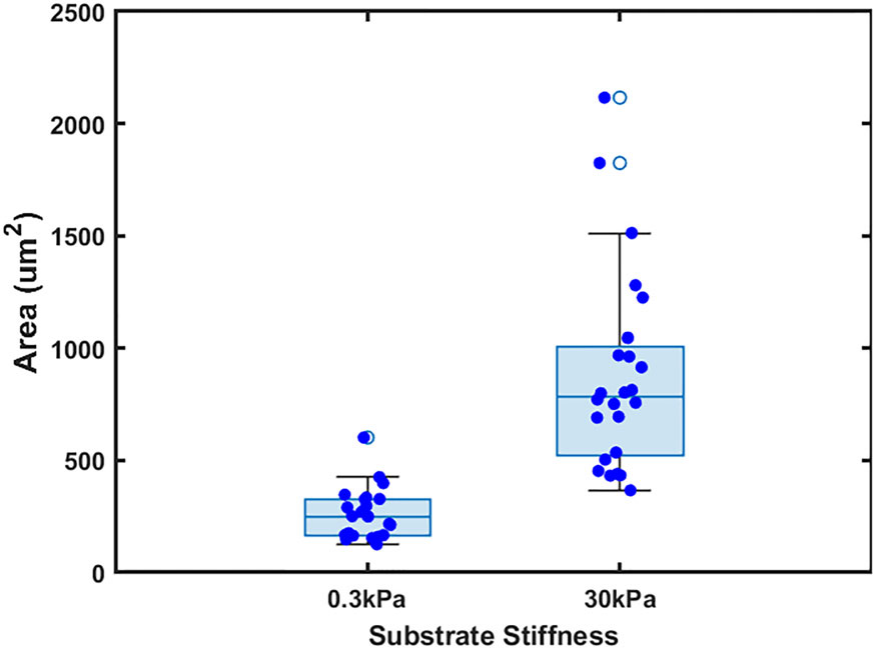
Quantification of the projected area of PDAC‐A cells cultured on soft and stiff substrates from the confocal images of phalloidin‐stained cells. Cells exhibited a more rounded and less spread morphology on soft substrates, whereas they appeared more flattened and well‐spread on stiff substrates, resulting in a higher projected cell area on stiff substrates. The mean cell area on soft substrates is 258.52 ± 24 µm² (Mean ± SE), compared to 879.01 ± 91 µm² (Mean ± SE) on stiff substrates. A Student's t‐test comparing the projected area of cells on soft (N = 23) and stiff (N = 24) substrates, where N is the number of cells, showed a statistically significant difference (p<0.0001).

### Time Considerations

#### Hydrogel preparation

This process requires 24 hours. NaOH treatment requires 1 hour and drying needs 20 minutes. Bind‐Silane treatment can be done in 7 minutes, followed by drying for 20 minutes. Hydrogel polymerization needs 30 minutes. Sulfo‐SANPAH treatment and UV exposure require 15 minutes. Fibronectin coating is done overnight.

#### Live‐cell staining

This staining process needs 6 hours, with PBS washing and cell seeding requiring 5 minutes each. Cell adherence requires 4–6 hours, and fluorescent dye staining needs 30 minutes.

#### Cell fixation and immunostaining

This process requires 24 hours. Formaldehyde fixation requires 20 minutes, Triton X‐100 permeabilization requires 15 minutes, and phalloidin staining needs 20 minutes. Mounting medium application and drying are done overnight.

### Author Contributions

**Shatruhan Singh Rajput**: Conceptualization; investigation; writing—original draft; methodology; writing—review and editing; formal analysis; validation; visualization. **Osian W. Jones**: Conceptualization; investigation; writing—original draft; methodology; validation; visualization; writing—review and editing; formal analysis. **Laura M. Machesky**: Conceptualization; funding acquisition; writing—review and editing; project administration; supervision.

### Conflict of Interest

The authors declare no conflict of interest.

## Data Availability

The data that support the protocol are available from the corresponding author upon reasonable request.
